# Vascular deficiency of *Smad4* causes arteriovenous malformations: a mouse model of Hereditary Hemorrhagic Telangiectasia

**DOI:** 10.1007/s10456-018-9602-0

**Published:** 2018-02-19

**Authors:** Angela M. Crist, Amanda R. Lee, Nehal R. Patel, Dawn E. Westhoff, Stryder M. Meadows

**Affiliations:** Cell and Molecular Biology Department, Tulane University, New Orleans, LA 70118 USA

**Keywords:** Smad4, Arteriovenous malformations (AVM), Hereditary hemorrhagic telangiectasia  (HHT), Vegfr2, TGFβ

## Abstract

Hereditary hemorrhagic telangiectasia (HHT) is an autosomal dominant vascular disorder that leads to abnormal connections between arteries and veins termed arteriovenous malformations (AVM). Mutations in TGFβ pathway members *ALK1*, *ENG* and *SMAD4* lead to HHT. However, a *Smad4* mouse model of HHT does not currently exist. We aimed to create and characterize a *Smad4* endothelial cell (EC)-specific, inducible knockout mouse (*Smad4*^f/f^;*Cdh5*-Cre^ERT2^) that could be used to study AVM development in HHT. We found that postnatal ablation of *Smad4* caused various vascular defects, including the formation of distinct AVMs in the neonate retina. Our analyses demonstrated that increased EC proliferation and size, altered mural cell coverage and distorted artery–vein gene expression are associated with *Smad4* deficiency in the vasculature. Furthermore, we show that depletion of *Smad4* leads to decreased *Vegfr2* expression, and concurrent loss of endothelial *Smad4* and *Vegfr2* in vivo leads to AVM enlargement. Our work provides a new model in which to study HHT-associated phenotypes and links the TGFβ and VEGF signaling pathways in AVM pathogenesis.

**Electronic supplementary material:**

The online version of this article (10.1007/s10456-018-9602-0) contains supplementary material, which is available to authorized users.

## Introduction

Hereditary hemorrhagic telangiectasia (HHT) is an autosomal dominant vascular disorder that affects 1 in 5000 people worldwide [1, 2]. HHT patients commonly exhibit: spontaneous, recurring nosebleeds; small lesions on mucous membranes called telangiectasias; and/or larger visceral lesions known as arteriovenous malformations (AVMs) [3, 4]. AVMs, which are direct connections between arteries and veins, are most commonly found in major organs such as the brain, liver or lungs. These lesions present a serious health risk and can lead to decreased quality of life and/or early death due to hemorrhaging, stroke and aneurysms [3, 5–8].

Approximately 85% of HHT cases are linked to heterozygous loss-of-function mutations in the transforming growth factor beta  (TGFβ)  cell surface receptors activin receptor-like kinase 1 (*ALK1*, HHT2) or endoglin (*ENG*, HHT1) [9, 10]. A small subset of HHT patients (~ 4%) exhibit haploinsufficiency of Mothers against decapentaplegic homolog 4 (*SMAD4*, JP/HHT) and commonly present with juvenile polyposis syndrome (JP) [11, 12]. SMAD4 is a transcription factor found in nearly all cell types [13, 14], where it serves as the central conduit through which canonical TGFβ signaling proceeds, including ALK1 and ENG signaling [15]. However, despite the key role of SMAD4 in the TGFβ pathway, the mechanisms by which it contributes to HHT pathogenesis remain unknown. In fact, virtually all HHT animal studies have focused on the *Alk1* and *Eng* receptor interface of the TGFβ signaling pathway, whereby endothelial loss of *Alk1*, or *Eng* or blockade of the TGFβ pathway via *Bmp9/10* ligand-blocking antibodies results in HHT-associated phenotypes [16–26]. What little we know about the in vivo role of SMAD4 in the vasculature comes from embryonic studies. These studies revealed that SMAD4 plays a critical role in blood vessel remodeling and maturation [27], integrity of the blood-brain barrier endothelium [28] and regulating coronary artery size [29]. Conversely, nothing is known about SMAD4 function in the postnatal vasculature as homozygous loss of *Smad4* is embryonic lethal [27]. Therefore, due to limited information on how SMAD4 contributes to the developing endothelium, it is unclear how SMAD4 defects lead to HHT phenotypes, such as AVM formation.

In order to better understand SMAD4’s contribution to HHT pathogenesis, we created an inducible, endothelial cell (EC)-specific *Smad4* knockout mouse model (referred to as *Smad4*-iECKO). We find that induced deletion of *Smad4* leads to various vascular defects including the formation of AVMs. In addition, we show that SMAD4 influences EC proliferation, EC size, mural cell coverage and artery–vein gene expression. Utilizing this new *Smad4*-iECKO model, we found that deletion of *Smad4* leads to decreased levels of vascular endothelial growth factor receptor 2 (VEGFR2) expression. Furthermore, concurrent loss of endothelial *Smad4* and *Vegfr2* in vivo leads to an increased AVM severity. This work provides a new model for the HHT field and presents evidence that the TGFβ and VEGF pathways may be linked in AVM pathogenesis.

## Results

### EC-specific deletion of *Smad4* causes multiple vascular defects, including AVM formation

**Fig. 1:**
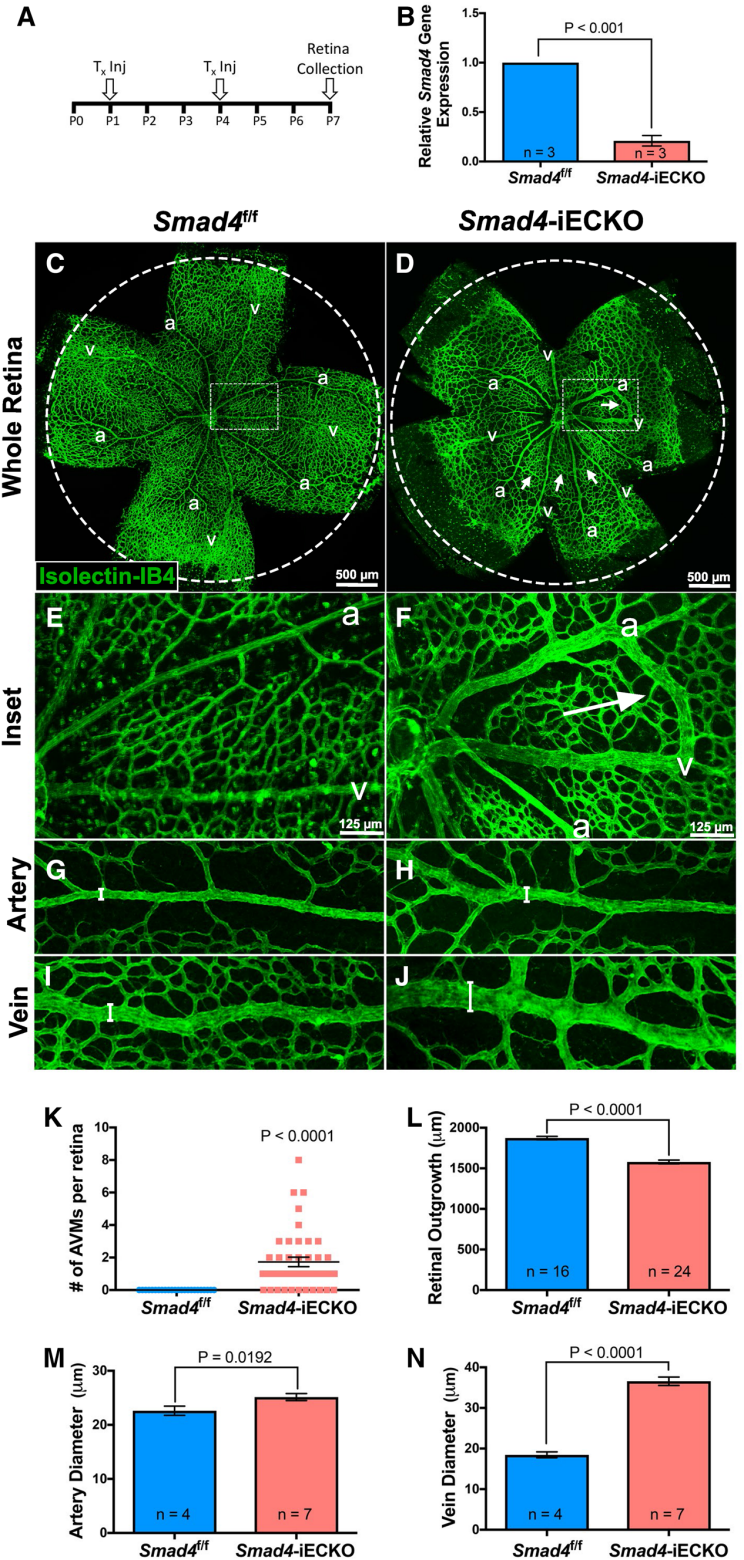
Multiple vascular defects, including AVMs, are associated with endothelial loss of *Smad4*. **A** Schematic outlining tamoxifen (Tx) injections in *Smad4*^f/f^ (control) and *Smad4*^f/f^;*Cdh5*-Cre^ERT2^ (hereafter referred to as *Smad4*-iECKO) neonate pups. **B** qPCR using RNA from freshly isolated retinal endothelial cells shows an 80% reduction in *Smad4* transcript levels (normalized to *Cd31*). **C–J** Confocal images of *Smad4*^f/f^ and *Smad4*-iECKO P7 retinas stained with isolectin-IB4 (IB4, green) to mark the retinal vasculature. **C**, **D** *Smad4*^f/f^ and *Smad4*-iECKO whole retina images illustrate the presence of AVMs (arrows) and reduced vascular outgrowth in *Smad4*-deficient genetic backgrounds. Dotted circles represent outgrowth of the control retina. Scale bar: 500 µm. **E**, **F** Magnified images of dotted boxes in **C** and **D** show normal separation of arteries and veins in control retinas, while *Smad4* mutants contained obvious AVMs (arrow). Scale bar: 125 µm. **G**, **H** Close-up views of a *Smad4*^f/f^ artery versus a *Smad4*-iECKO artery. Note the increased diameter of *Smad4* mutant arteries. **I**, **J** Close-up views of a *Smad4*^f/f^ vein versus a *Smad4*-iECKO vein. Note the increased diameter of *Smad4* mutant veins. **K** Quantification of the number of AVMs per retina in *Smad4* control and mutant backgrounds. **L** *Smad4*-iECKO mutants exhibited reduced vascular outgrowth toward the retinal periphery. Outgrowth was measured by taking the distance from the optic nerve to the outermost vessel in the vascular front. Four measurements were taken per retina (one on each retinal leaflet). **M**, **N** Graphs showing increased artery (**M**) and vein (**N**) diameters in *Smad4*-iECKO retinas compared to *Smad4*^f/f^ controls. Three points were measured on each vessel type (proximal, medial and distal from the optic nerve), and three arteries and veins were randomly chosen and measured per retina. *a* arteries, *v* veins. All Fig. 1 statistics include *Smad4* mutant retinas with and without AVMs, and areas around and separate from AVMs

To characterize SMAD4 function in the postnatal vasculature, *Cdh5*-Cre^ERT2^ [30] and conditional *Smad4*-floxed (Smad4^f/f^) [31] mouse lines were utilized to generate homozygous *Smad4*-inducible, endothelial-specific knockout mice (*Smad4*-iECKO). Tamoxifen (Tx) injections were administered at postnatal day 1 (P1) and P4 to activate Cre-mediated deletion of *Smad4* in the endothelium (Fig. 1A). A majority of mice died around P8–P9, likely due to respiratory distress caused by defects in the lung vasculature (Fig. S1A–B′), similar to *Alk1*-deficient neonate mice [20]. Therefore, we utilized P7 retinas to assess blood vessel development, as the retina is a tractable system for identifying vascular defects, including AVMs, and has been used to study *Alk1* and *Eng* mouse models of HHT.

To confirm *Smad4* deletion in the endothelial lineage, quantitative PCR (qPCR) was performed using RNA from P7 *Smad4*^f/f^ (control) and *Smad4*-iECKO isolated retinal ECs which revealed an ~ 80% reduction in *Smad4* mRNA transcripts (Fig. 1B). In addition, using a *Rosa26*-EYFP transgenic reporter line [32] we confirmed that Cre-recombinase was specifically expressed in blood vessels, while absent in control blood vessels (Fig. S1C–D′). These data demonstrated efficient and specific *Smad4* knockdown in the ECs of *Smad4*-iECKO retinas.

In order to assess the effects of *Smad4* depletion on vascular development, *Smad4*^f/f^ control and *Smad4*-iECKO P7 retinas were labeled with the vascular marker Isolectin-IB4. We observed numerous arteriovenous malformations (AVMs) in the retinas of *Smad4* mutants (Fig. 1C–F), similar to those identified in *Alk1*- and *Eng*-deficient mice [17, 18, 20, 24, 33, 34]. Approximately 82% of our *Smad4* mutants had AVMs, whereas AVMs were absent in all controls. Multiple AVMs were seen in 52% of *Smad4*-iECKO mice with an average of 1.732 AVMs per mutant retina (Fig. 1K). AVMs varied in morphology but were easily identifiable because the shunts appeared grossly enlarged in comparison with normal capillaries (Fig. 1C–F and Fig. S2A–H). AVMs were almost always located near the center of the retina, likely due to blood flow patterns in HHT models as previously described [35]. In *Smad4*-iECKO mice, AVMs form around P5 (data not shown) and either did not form or were smaller if Tx was administered after P1 (Fig. S2I–K).

Loss of *Smad4* also caused a noticeable reduction in vascular outgrowth toward the retinal periphery (Fig. 1C, D, L). For this reason and because *Alk1* mutant zebrafish exhibit EC migratory defects [36], we aimed to further assess *Smad4* function on EC migration in vitro. We generated stable C166 mouse EC lines that expressed either nonsilencing-shRNAs or Smad4-shRNAs. In comparison with nonsilencing-shRNA, Smad4-shRNA C166 cells showed an approximately 60% reduction in levels of *Smad4* transcripts and a diminished capacity to migrate and repopulate wounds in a scratch assay (Fig. S3A–D).

Although outgrowth was stunted, the number of tip cells was not significantly changed in *Smad4* mutant retinas (data not shown). Similarly, quantification of vascular densities showed no statistical differences on average (data not shown). This is likely due to the high variability between mutants, as we observed some mutants that displayed significant increases in density in the tip region, while others were indistinguishable from controls (Fig. S4). Although there was variability among vascular density of mutants, one consistent phenotype was that of increased artery and vein diameters (Fig. 1G–J, M, N). Increases in vessel diameter and stunted outgrowth were also even seen in the ~ 20% of mutants that did not express AVMs, suggesting that these events may precede AVM development.

Collectively, these results demonstrate that *Smad4* is required for proper vascular growth and vessel morphology in the postnatal retina. Furthermore, the presence of AVMs verifies that loss of *Smad4* in mice recapitulates phenotypes associated with HHT patients, thus making this a suitable model for studying *Smad4 *mechanisms of HHT.

### *Smad4* depletion causes increases in EC proliferation and size

**Fig. 2:**
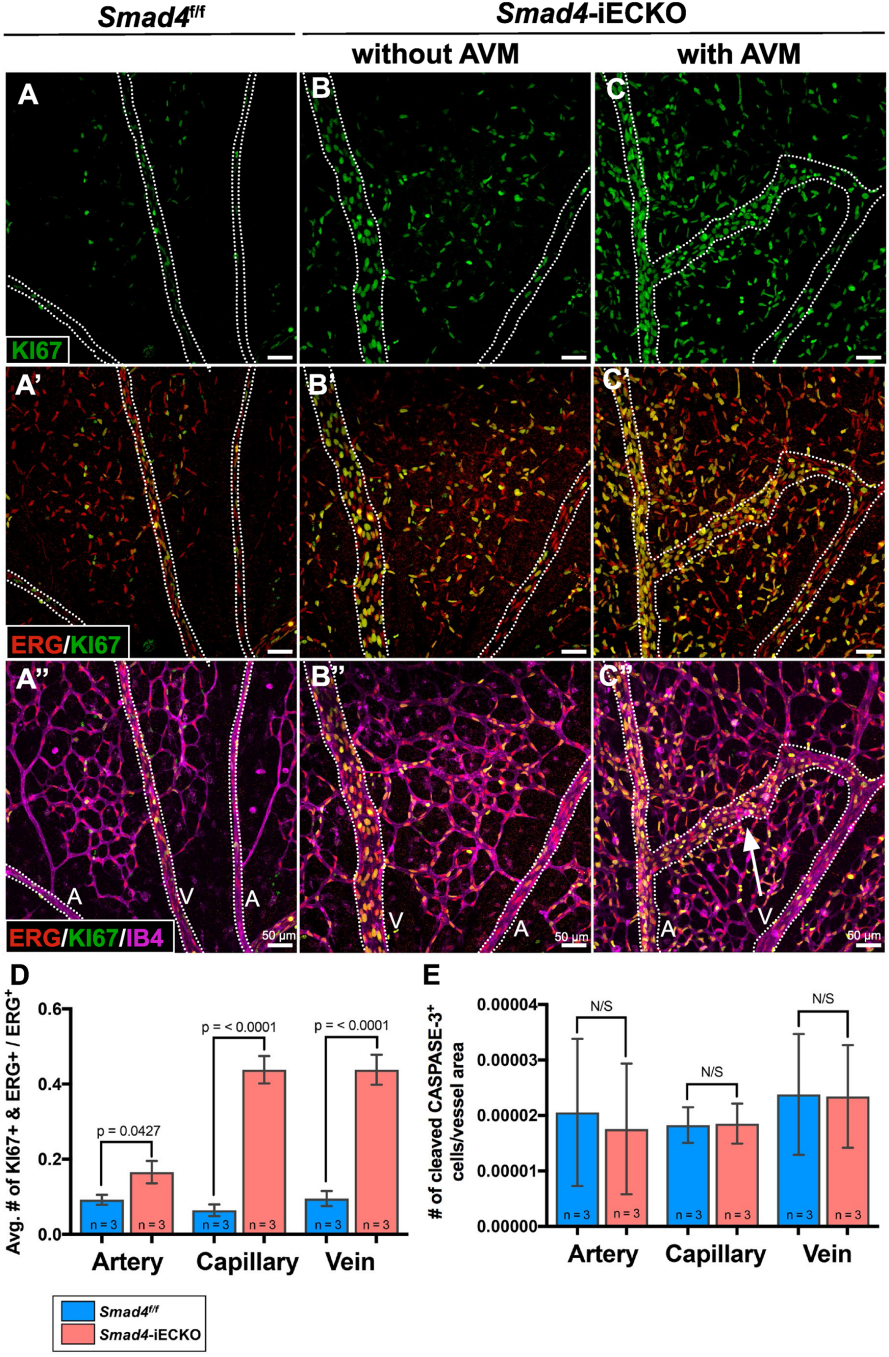
Increased cell proliferation in *Smad4*-deficient ECs. **A**–**C**” Close-up views of *Smad4*^f/f^ and *Smad4*-iECKO P7 retina stained for KI67 (green), ERG (red) and Isolectin-IB4 (magenta; IB4). *a* arteries, *v* veins and AVM (white arrow) in **A**–**C**’ are outlined by dotted lines. Scale bars = 50 µm. **D** *Smad4* deficiency leads to an increase in proliferating ECs (KI67^+^ and ERG^+^) in *Smad4*-iECKO arteries, capillaries and veins. Three fields of view were counted in each sample. **E** Loss of *Smad4* reveals no change in the number of apoptotic cells as assessed by cleaved CASPASE-3 fluorescence in the P7 retinal vasculature. Three fields of view were counted in each sample. Sample size (*n*) indicates independent biological samples

To determine whether vessel enlargement was caused by an increase in EC proliferation, we examined both *Smad4* control and mutant retinas immunolabeled for the proliferation-associated protein, KI67, and the EC-specific nuclear marker, ERG. Proliferating ECs, which are defined as both KI67 and ERG positive, were quantified in the arteries, veins and capillaries (Fig. 2A–D). We found significant increases in proliferation in all vessel types, with the most drastic changes seen in the capillaries and veins. Increases in proliferation occurred in vascular regions with and without AVMs, indicating that these increases are attributable to reduction in SMAD4 levels and not an effect of AVM formation itself. Conversely, staining for the apoptotic marker cleaved CASPASE-3 revealed no changes in cells undergoing apoptosis between control and *Smad4*-iECKO retinal blood vessels (Fig. 2E). These data indicated that increased rates of EC proliferation, at least in part, are responsible for the increased vessel sizes in *Smad4*-iECKO retinas.

**Fig. 3:**
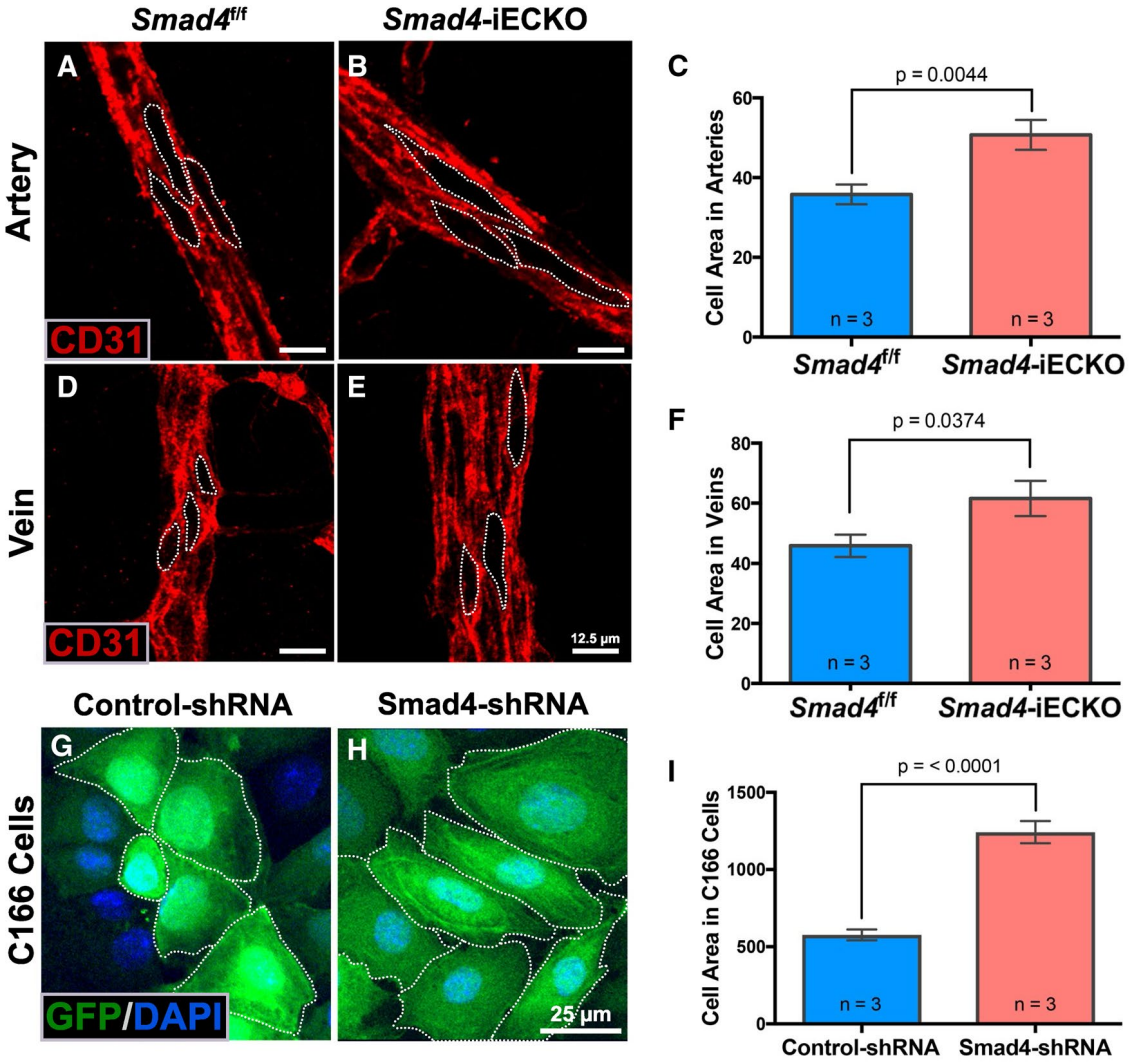
Loss of *Smad4* causes an increased EC size. **A**–**E** *Smad4*^f/f^ and *Smad4*-iECKO P7 retinal arteries (**A**, **B**) and veins (**D**, **E**) immunolabeled for CD31/PECAM (red) to mark EC–EC boundaries revealed an increase in cell size upon loss of *Smad4*. Scale bar: 12.5 µm. **C**, **F** Quantification of EC areas within CD31-stained regions demonstrated enlarged ECs in both arteries and veins of *Smad4* mutants compared to controls. Sample size (*n*) indicates independent animals. Three ECs from each of three random arteries and veins were measured per retina. For these experiments, we did not categorize vessels as near or far from AVMs, and mutants with and without AVMs were included. **G**–**H** GFP- and DAPI-stained nonsilencing-shRNA and Smad4-shRNA C166 cells expressing an internal ribosome entry site (IRES) mediated GFP. Dotted lines mark the cell boundaries as distinguished by the cytoplasmic GFP. Scale bar: 25 µm. **I** Quantification of EC sizes showed significant increases in cell areas of Smad4-shRNA cells compared nonsilencing-shRNA C166 cells. For image quantification, three fields of view were used per sample and a minimum of three ECs was measured per field of view

Relatedly, recent reports indicated that loss of either *Eng* or* Smad4* leads to an increased EC size [25, 29], which could contribute to an increased artery and vein diameter and/or AVM formation. To determine whether *Smad4*-iECKO mice exhibited changes in cell size, we measured EC areas in each vessel type as marked by CD31-stained EC boundaries. *Smad4* deletion caused the cell area of arterial ECs to increase by 41%, while venous ECs increased by 34% compared to their respective control vessels (Fig. 3A–F). In addition, C166 ECs with Smad4-shRNA showed a 115% increase in cell area compared to nonsilencing-shRNA C166 ECs (Fig. 3G–I). Overall, these results are consistent with a recent study showing that loss of *Smad4* contributes to an increase in cell size in the developing coronary artery and in cultured ECs under high levels of shear stress [29]. Taken together, we conclude that a combination of an increased EC proliferation and an increased EC size contributes to the vessel enlargement phenotypes observed in *Smad4* mutants.

### Defective mural cell coverage in *Smad4*-iECKO mice

**Fig. 4:**
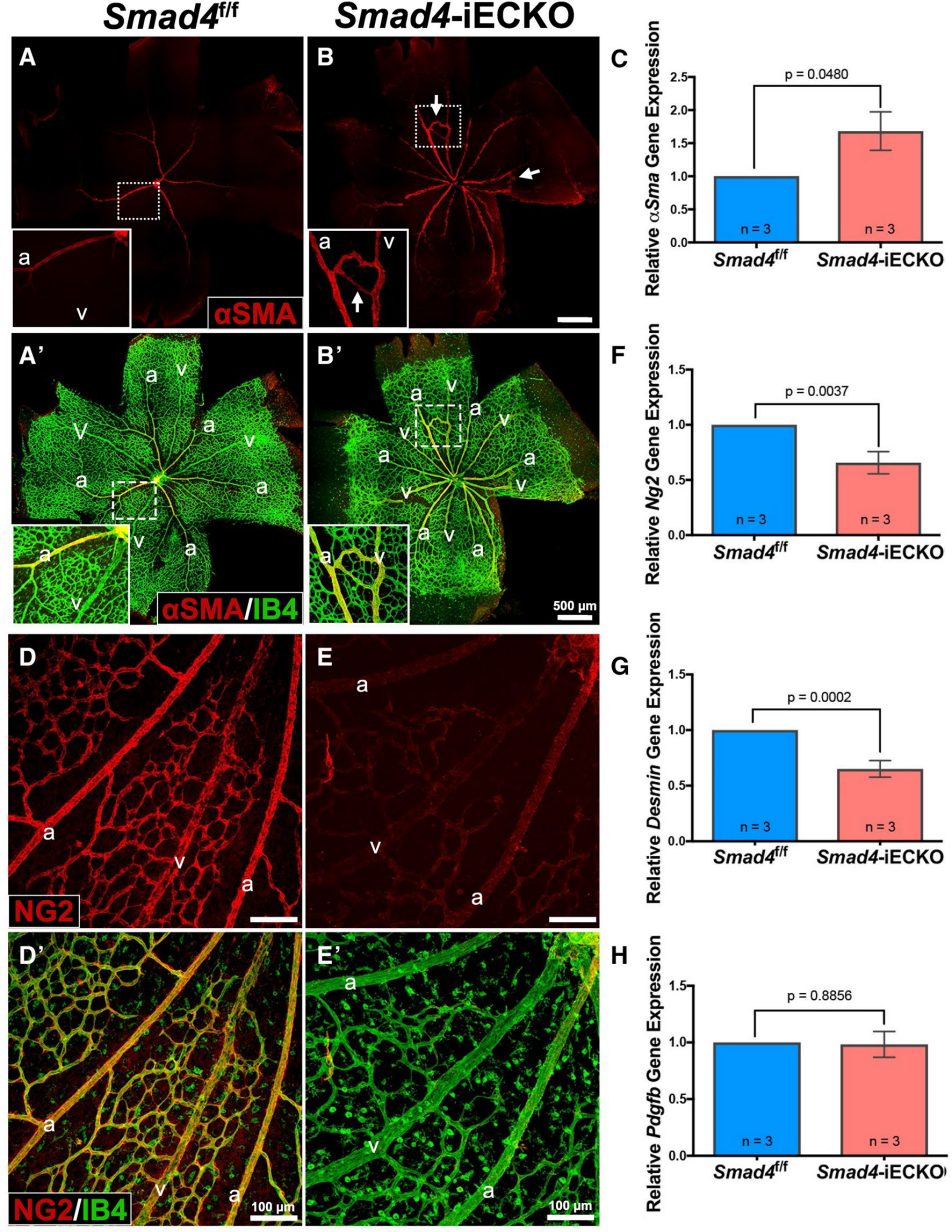
Altered mural cell coverage in *Smad4*-iECKO retinas. **A**–**B**’ Confocal analysis of alpha-smooth muscle actin (αSMA; red) and Isolectin-IB4 (IB4; green) revealed that *Smad4*^f/f^ retinas (*n* = 10) contain αSMA only on arteries, whereas *Smad4*-iECKO mice (*n* = 8) express αSMA on both arteries and veins. Insets show close-up views of an artery and vein, with an AVM covered by αSMA in **B**, **B′** inset. AVMs are denoted by white arrows in **B**. **C** qPCR results on RNA isolated from *Smad4*^f/f^ and *Smad4*-iECKO P7 whole retinas confirm an increased *αSma* gene expression in Smad4 mutants. **D**–**E**’ Immunofluorescent staining of the pericyte marker neuron-glial antigen 2 (NG2; red) and IB4 (green) revealed a striking reduction in levels of NG2 in *Smad4*-iECKO mice (*n* = 9) compared to control (*n* = 10). **F**, **G** qPCR analysis of *Ng2* and *Desmin* (another pericyte marker) transcript levels in P7 *Smad4*^f/f^ and *Smad4*-iECKO whole retinas verifies loss of pericytes in *Smad4*-deficient backgrounds. **H** qPCR analysis of secreted ligand *Pdgfb* transcript levels in P7 *Smad4*^f/f^ and *Smad4*-iECKO whole retinas remains unchanged. *a* arteries, *v* veins. The number of independent biological samples is shown as (*n*) on bar graphs, and all qPCR samples were normalized to the housekeeping gene *Odc*

*Alk1* and *Eng* models of HHT have previously noted several changes in mural cell coverage of retinal blood vessels. For instance, vascular smooth muscle cells (vSMCs) were found to accumulate ectopically around veins [17, 20, 24]. In *Smad4*-iECKO mice, we also observed strong, ectopic expression of alpha-smooth muscle actin (αSMA) protein around the AVMs and veins compared to control retinas which only exhibit αSMA on arteries at P7 (Fig. 4A–B’). Moreover, qPCR analysis demonstrated increased *αSma* transcript levels in *Smad4*-iECKO retinas compared to *Smad4*^f/f^ controls (Fig. 4C).

Conversely, AVMs are associated with a reduction in pericyte coverage [20, 35]. *Smad4* deficiency has been shown to affect EC–pericyte interactions resulting in loss of pericyte coverage in the developing brain vasculature [28]. To test whether this relationship exists in *Smad4*-iECKO retinas, we investigated pericyte coverage using an anti-neuron-glial antigen 2 (NG2) antibody. We found that compared to controls, *Smad4* mutant retinas exhibit a marked reduction in NG2 protein accumulation in the retinal vasculature (Fig. 4D–E'). Furthermore, qPCR analysis on whole retina samples verified that *Ng2* and *Desmin* (a pericyte marker) mRNA levels are significantly diminished when *Smad4* is deleted (Fig. 4F, G). Since the platelet-derived growth factor (PDGF) signaling pathway plays a significant role in recruiting pericytes to blood vessels [37], we assessed whether changes in expression of the endothelial-secreted PDGFB ligand could account for the loss of pericyte coverage in *Smad4* mutants. qPCR results showed that *Pdgfb* transcript levels are similar in control and *Smad4*-iECKO whole retinas (Fig. 4H), suggesting that other factors are responsible for the reduced pericyte presence in *Smad4*-deficient retinas. Overall, our results are consistent with other HHT models in that vSMC coverage inappropriately extends to AVMs and veins, while pericyte coverage is reduced in a *Smad4*-deficient background.

### Artery–vein identity is disrupted in the absence of *Smad4*

**Fig. 5:**
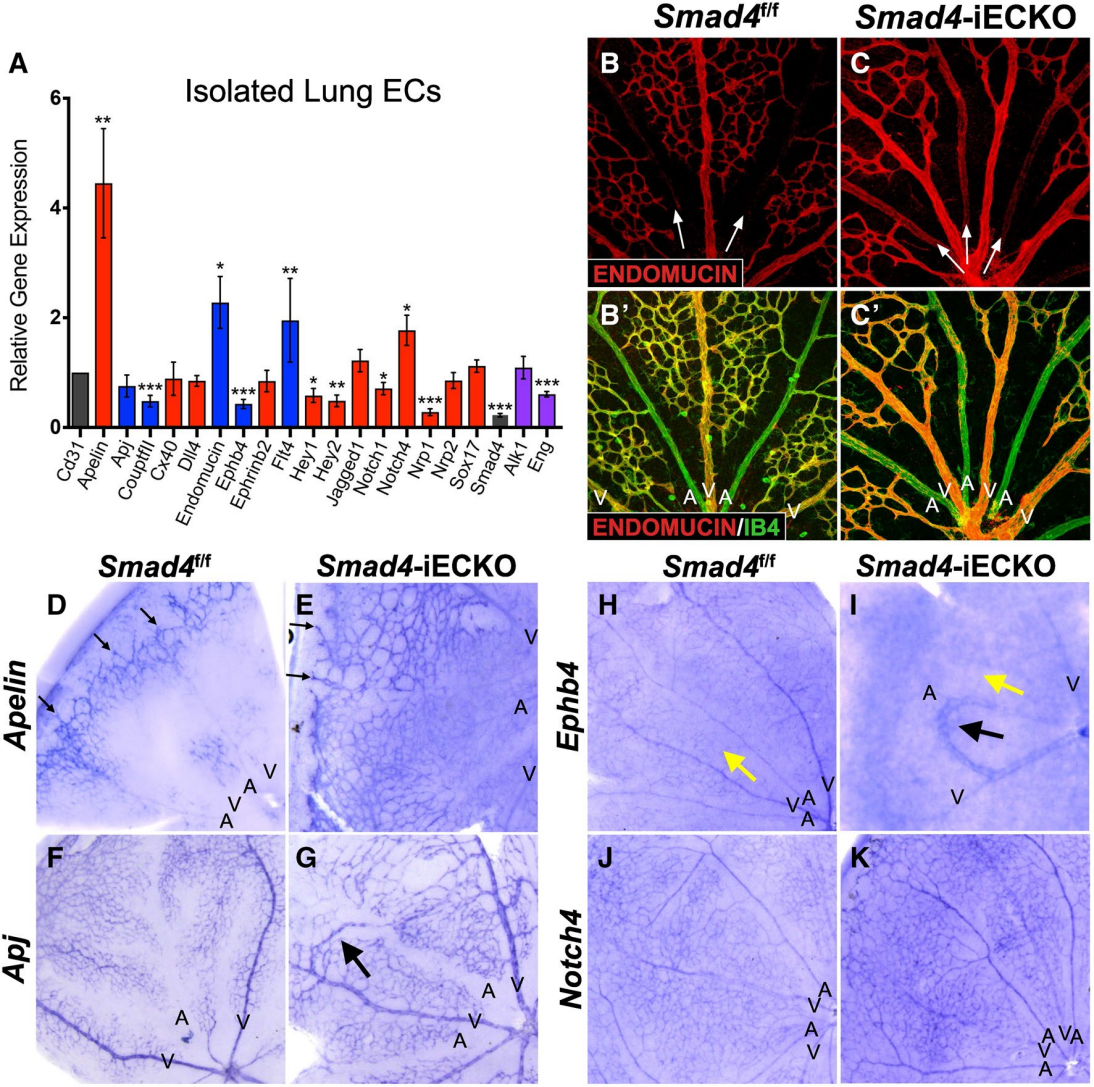
*Smad4*-iECKO mice display alterations in artery and vein gene expression profiles. **A** qPCR analysis of 12 arterial (red), 5 venous (blue) and 2 HHT (purple)-associated genes on isolated, cultured lung ECs (iLECs) from P7 *Smad4*^f/f^ (*n* = 3) and *Smad4*-iECKO (*n* = 3) mice revealed altered AV- and HHT-associated gene expression. All transcripts were normalized to *Cd31/Pecam* mRNA levels (*p < 0.05; **p < 0.005; ***p ≤ 0.0001). **B**–**C**’ Qualitative evaluation of *Smad4* control (*n* = 6) and mutant (*n* = 6) P7 retinas revealed increased levels of ENDOMUCIN protein (red) in arteries of *Smad4*-iECKO mice (white arrows; IB4, green). **D**–**K** In situ hybridization analysis of various artery–vein mRNAs substantiates qPCR results from iLECs. **D**, **E** *Apelin* mRNA is localized to the vascular tip cells (black arrows) and arteries of *Smad4*^f/f^ mice (*n* = 3); however, in *Smad4*-iECKO mice *Apelin* is also robustly and ectopically expressed in the capillaries and veins (*n* = 3). **F**–**G** *Apj* expression remains unchanged in *Smad4*^f/f^ (*n* = 3) and *Smad4*-iECKO (*n* = 3) littermate retinas. Arrow in **G** points to an AVM expressing *Apj*. **H**–**I** *Ephb4* mRNA is expressed strongly in the veins and moderately in the arteries and capillaries (yellow arrow) of *Smad4*^f/f^ retinas (*n* = 3). In *Smad4*-iECKO retinas, *Ephb4* is largely absent in the capillary beds (yellow arrow) and in most arteries, excluding the artery connected to the AVM (black arrow) (*n* = 3). Note that *Ephb4* mRNA is expressed in the AVM. **J**–**K** *Notch4* mRNA expression patterns remain the same in *Smad4*^f/f^ (*n* = 3) and *Smad4*-iECKO (*n* = 3) littermate retinas, but overall levels are increased in all vessel types of *Smad4* mutants. *a* arteries, *v* veins, *n* the number of independent biological samples

The presence of vSMCs on *Smad4*-deficient veins suggested that these vessels may have acquired an arterial-like identity. Interestingly, alterations in artery and vein (AV) gene expression have been reported in HHT models [17, 20, 38, 39] and in non-HHT-associated AVMs triggered by disruptions in NOTCH pathway signaling components [40, 41]. Therefore, we characterized a number of AV identifiers, including NOTCH pathway members, in our *Smad4*-iECKO model by performing qPCR on isolated lung endothelial cells (iLECs) from *Smad4* mutants and their control littermates (Fig. 5A). We found a significant reduction in the venous-associated markers *CouptfII* and *Ephb4* and arterial markers *Hey1, Hey2, Notch1* and *Nrp1*. Conversely, mRNA levels of the venous markers, *Endomucin* and *Flt4*, and the arterial markers *Apelin* (also associated with vascular tip cells) and *Notch4* were upregulated in *Smad4*-iECKO iLECs. The following markers remained unchanged between *Smad4* control and mutants: *Apj, Cx40, Dll4, Ephrinb2, Jagged1, Nrp2* and *Sox17*.

To further confirm changes in AV gene expression, we used immunofluorescent staining and in situ hybridization techniques on *Smad4*^f/f^ and *Smad4*-iECKO retinas. At P7, ENDOMUCIN is largely absent from control retinal arteries (Fig. 5B); however, in *Smad4* mutants, we observed distinct protein expression in the arteries (Fig. 5C), suggesting that increased *Endomucin* mRNA levels in iLECs (Fig. 5A) might be due to enhanced expression in arteries. Analysis of *Apelin* mRNA showed robust, ectopic expression in the retinal veins and capillaries of *Smad4* mutants compared to controls, which completely lacked expression in these vessels (Fig. 5D, E). On the other hand, the apelin receptor, *Apj*, was present in the AVM but showed no noticeable changes in mRNA expression between* Smad4* control and mutant retinas consistent with our qPCR results (Fig. 5F, G). Examination of *Ephb4* revealed the loss of transcripts in the capillaries and arteries, although *Ephb4* mRNA remained in the veins (Fig. 5H, I). Interestingly, even though overall levels of *Ephb4* mRNA were reduced, *Ephb4* was still noticeably expressed within the AVM and the artery connected in the AVM. Analysis of *Notch4* showed no changes in localization of transcripts between *Smad4* control and mutant retinas; however, levels of *Notch4* appeared markedly higher in *Smad4*-iECKO retinas (Fig. 5J, K). In all, the whole retina staining results are consistent with the qPCR analysis performed on iLECs. This also revealed the importance of examining both qPCR levels in the whole vasculature as well as localization changes, as some markers are gained in specific vascular beds (*Apelin*), while others are lost (*Ephb4*). More so, some markers can increase in levels without changing localization (*Notch4*), while others remain the same but are expressed in the AVM (*Apj*). Therefore, we conclude that loss of *Smad4* in the endothelium alters AV gene expression and as other groups have suggested may play a contributing role in the formation of AVMs due to disruptions in vessel identity [41].

In addition, we quantified the transcript levels of *Alk1* and *Eng* in *Smad4* control and mutant iLECs, as previous reports have shown that genetic knockdown of one receptor can lead to changes in expression of the other in vivo [17, 20, 24]. Our qPCR results indicate a significant loss in expression of *Eng* in *Smad4*-depleted ECs; however, no changes in *Alk1* expression were observed (Fig. 5A).

### Loss of *Vegfr2* enhances *Smad4* mutant phenotypes

**Fig. 6:**
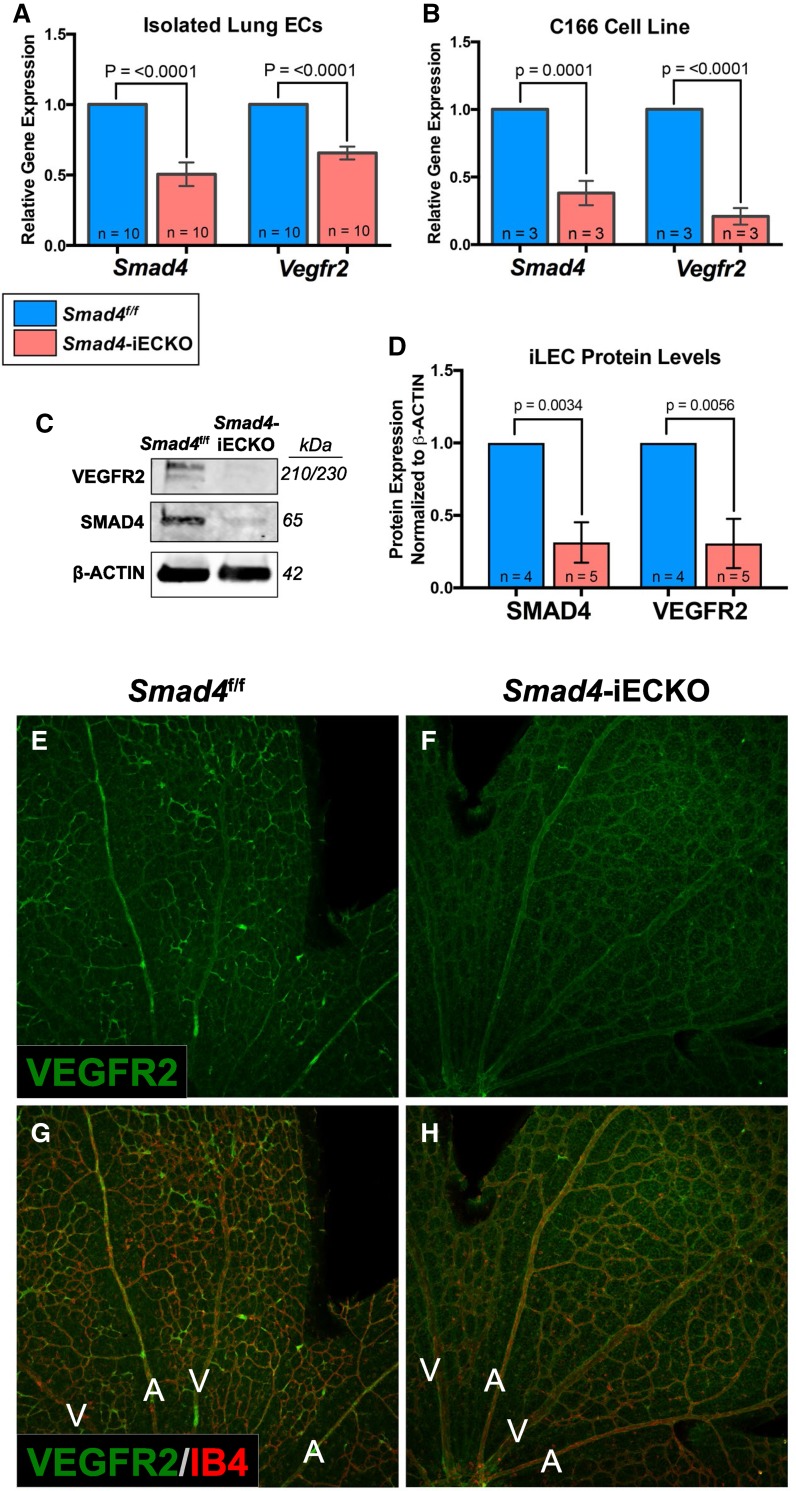
Postnatal ablation of *Smad4* leads to reduced levels of *Vegfr2*. **A**, **B** qPCR analysis of *Smad4* and *Vegfr2* mRNA levels in isolated lung endothelial cells (**A**, normalized to *Cd31*) and C166 cells (**B**, normalized to *Odc*) reveals a significant reduction of *Vegfr2* transcripts when *Smad4* is knocked down. **C** Representative western blot shows reductions in SMAD4 and VEGFR2 proteins in *Smad4*-iECKO isolated lung ECs compared to *Smad4*^f/f^ controls. **D** Western blot quantifications verify decreased VEGFR2 protein levels in *Smad4*-depleted iLECs. **E**–**F** Immunofluorescent staining of IB4 (red) and VEGFR2 (green) on P7 *Smad4*^f/f^ (*n* = 3) and *Smad4*-iECKO (*n* = 4) retinas indicates reduced expression of VEGFR2 protein in *Smad4*-depleted blood vessels. Scale bar represents 200 µm. Sample size (*n*) represents the number of independent biological samples

Recent investigations in *Alk1* and *Eng* HHT models indicated a potential link with vascular endothelial growth factor receptor 2 (*Vegfr2*) [24, 34, 42], a major signaling component in the VEGF pathway. In order to determine whether *Smad4* and *Vegfr2* are associated in AVM pathogenesis, we first assessed whether *Vegfr2* mRNA levels were altered in our *Smad4*-iECKO background. qPCR analysis on cultured iLECs demonstrated that, in comparison with controls, *Vegfr2* mRNA levels are reduced by approximately 40% in *Smad4*-iECKO mice (Fig. 6A). However, no significant changes in *Vegfr2* transcript levels were observed between Tx-injected *Smad4*^f/f^ control and* Smad4*^f/+^;*Cdh5*-Cre^ERT2^ iLECs, even though *Smad4 *mRNA levels were reduced by approximately 50% in the heterozygous mutants (data not shown). Similarly, quantification in Smad4-shRNA C166 endothelial cells also showed a relationship whereby *Vegfr2* transcripts were significantly reduced compared to nonsilencing-shRNA controls (Fig. 6B). Moreover, expression of VEGFR2 protein was dramatically diminished in *Smad4*-deficient iLECs (Fig. 6C, D) and appeared reduced throughout all retinal blood vessels in *Smad4* mutants as compared to controls (Fig. 6E–H). In all, these data demonstrated that impaired SMAD4 function leads to significantly reduced expression of *Vegfr2* mRNA and protein.

**Fig. 7:**
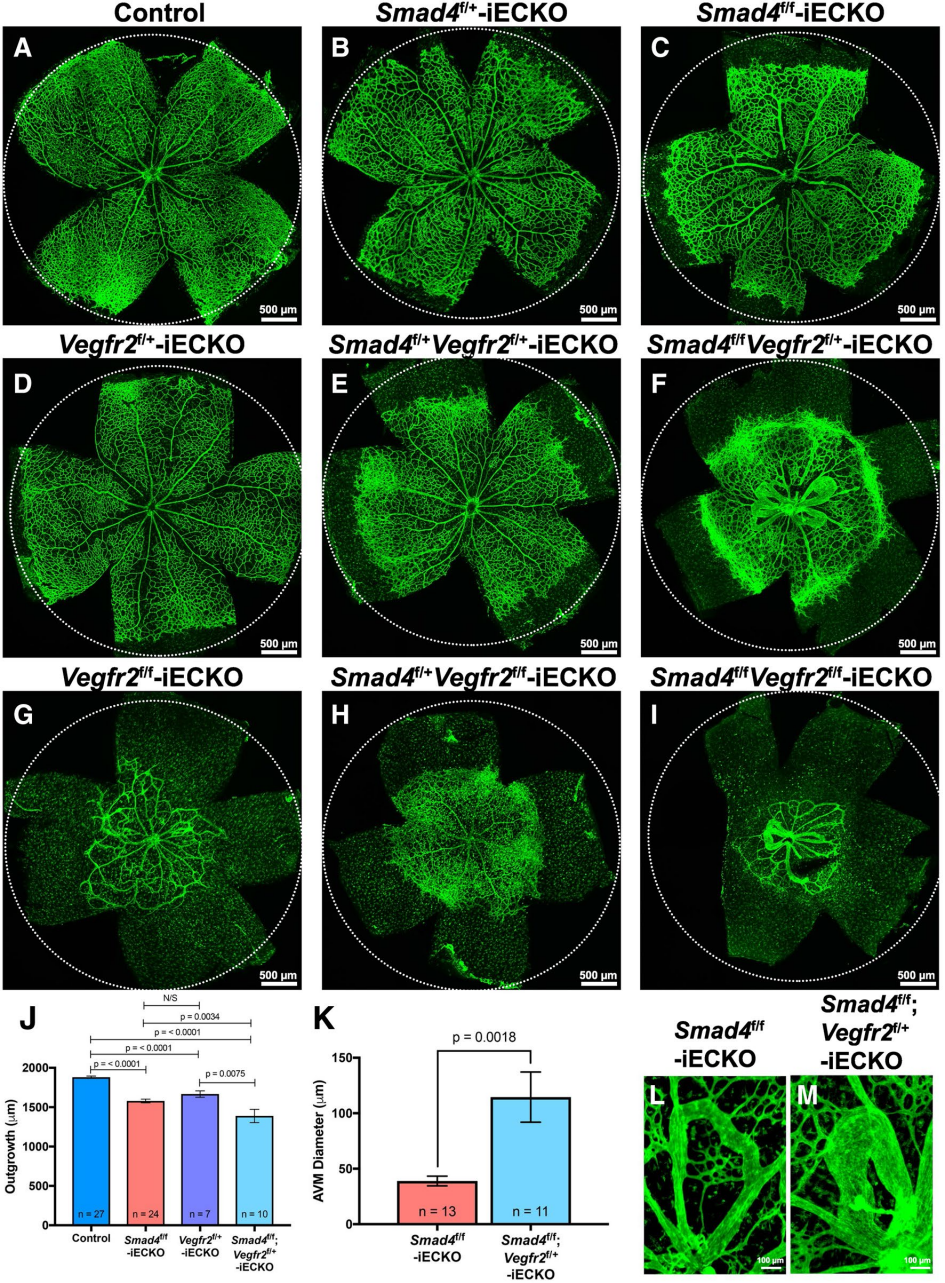
Postnatal ablation of *Smad4* and *Vegfr2* leads to enhanced AVM severity in the mouse retina. **A**–**I** Isolectin-IB4 staining  (green) of P7 murine retinas from the following genotypes: **A** *Smad4*^f/f^ control (*n* = 27), **B** *Smad4*^f/+^-inducible endothelial cell knockout (iECKO) control (*Smad4*^f/+^;*Cdh5*-Cre^ERT2^) (*n* = 5), **C** *Smad4*^f/f^-iECKO (*n* = 24), **D** *Vegfr2*^f/+^-iECKO control (*n* = 7), **E** *Smad4*^f/+^;*Vegfr2*^f/+^-iECKO (*n* = 5), **F** *Smad4*^f/f^;*Vegfr2*^f/+^-iECKO (*n* = 14), **G** *Vegfr2*^f/f^-iECKO (*n* = 3), **H** *Smad4*^f/+^;*Vegfr2*^f/f^-iECKO (*n* = 7) and **I** *Smad4*^f/f^;*Vegfr2*^f/f^-iECKO (*n* = 7). Dotted circles represent vascular outgrowth of the control retina in **A**. Scale bar represents 500 µm. **J** Quantification of vascular outgrowth in select genotypes revealed statistically significant decreases in *Smad4*^f/f^;*Vegfr2*^f/+^-iECKO retinas. **K** *Smad4*^f/f^;*Vegfr2*^f/+^-iECKO mice exhibit an increased AVM severity as measured by AVM diameter. **L, M** Close-up views comparing AVMs in *Smad4*-iECKO and *Smad4*^f/f^;*Vegfr2*^f/+^-iECKO retinas

Since our data indicated that *Vegfr2* expression decreases upon loss of* Smad4*, we hypothesized that the incremental losses of VEGFR2 in the *Smad4*-iECKO background would lead to enhanced HHT-like phenotypes during retinal vascular development. To test this possibility, we crossed our *Smad4*-iECKO mouse line to *Vegfr2*-floxed mice (*Vegfr2*^f/f^, referred to as *Vegfr2*^f/+^-iECKO or *Vegfr2*^f/f^-iECKO in the presence of *Cdh5*-Cre^ERT2^) [43]. The different genetic combinations of mice were given Tx at P1, and retinas were collected at P7. Similar to control *Smad4*^f/f^ mice lacking *Cdh5*-Cre^ERT2^, a single allelic deletion of *Smad4* (*Smad4*^f/+^-iECKO) had no noticeable effects on retinal vascular development, while *Vegfr2* (*Vegfr2*^f/+^-iECKO) retinas only showed a reduction in vascular outgrowth (Fig. 7A, B, D). On the other hand, the combined loss of a single allele of *Smad4* and *Vegfr2* (*Smad4*^f/+^;*Vegfr2*^f/+^-iECKO) resulted in increases in vascular density at the growing front (although inconsistent), which were never observed in *Smad4*^f/+^-iECKO or *Vegfr2*^f/+^-iECKO retinas, but were similar to *Smad4*^f/f^-iECKO retinas (compare Fig. 7 B–E and Fig. S4). More compellingly, loss of a single copy of the *Vegfr2* allele in the *Smad4*-iECKO background (*Smad4*^f/f^;*Vegfr2*^f/+^-iECKO) revealed dramatic vascular phenotypes beyond those observed in *Smad4*-iECKO mice alone (compare Fig. 7C and F). Vascular outgrowth was significantly inhibited, and the vascular front showed reliable and marked increases in density (Fig. 7J). Even more noticeable was the increased number and striking enlargement of AVMs. We quantified severity of AVMs by measuring the diameter of the AVM in *Smad4*-iECKO and *Smad4*^f/f^;*Vegfr2*^f/+^-iECKO mice. On average, *Smad4*^f/f^;*Vegfr2*^f/+^-iECKO AVMs were ~ 75 µm wider than *Smad4*-iECKO AVMs, suggesting that more blood is able to be shunted from artery to vein in these AVMs leading to a more severe phenotype (Fig. 7K–M). Together, these experiments implied that overall levels of VEGFR2 have an effect on severity of *Smad4*-mediated HHT phenotypes.

Interestingly, reciprocal experiments in *Vegfr2* null backgrounds showed slightly different results. As previously observed, complete loss of *Vegfr2* (*Vegfr2*^f/f^-iECKO) in the retina led to severe vascular defects, including an overall reduction in the vasculature with fewer vessels and a lack of definitive capillaries (compare Fig. 7A, G) [44]. Loss of a single allele of *Smad4* in the *Vegfr2* null background (*Smad4*^f/+^;*Vegfr2*^f/f^-iECKO) resulted in similar phenotypes, suggesting that heterozygous loss of* Smad4* had little affect on the overall vascular phenotype (Fig. 7H). In addition, combinatorial deletion of *Smad4* and *Vegfr2* (*Smad4*^f/f^;*Vegfr2*^f/f^-iECKO) led to AVM formation, but these retinas exhibited very little vascular coverage (Fig. 7I). This suggests that there is a threshold for which loss of *Vegfr2* becomes dominant to *Smad4* deletion, as was previously reported in *Alk1* mutants [24]. In conclusion, our results indicate that reduction in VEGF signaling may contribute to heightened HHT phenotypes during developmental angiogenesis.

## Discussion

Our studies are the first to report a *Smad4* animal model of HHT (*Smad4*-iECKO). We showed that endothelial loss of *Smad4* recapitulates vascular phenotypes seen in other HHT mouse models, particularly AVM formation. To better understand *Smad4*’s role in HHT pathogenesis, we performed a comprehensive characterization of *Smad4*-iECKO mice. Our results demonstrated that increased EC proliferation and size, alterations in mural cell coverage and disruption in AV gene expression are associated with *Smad4*-deficient blood vessels. We also provided evidence that loss of SMAD4 causes decreased VEGFR2 expression, and that loss of a single allele of *Vegfr2* in the *Smad4* null background leads to an increased severity of AVMs.

Considering *Smad4*’s centralized role in TGFβ signaling, we aimed to test the universality of our *Smad4*-iECKO model in relationship to HHT phenotypes. Consistent with previous reports, loss of TGFβ signaling through *Smad4* leads to vascular defects similar to those found in *Eng* and *Alk1* mouse retinal models (Fig. 1; summarized comparisons of HHT models in Table 1). For example, blood vessel enlargement in *Alk1* and* Eng *mutant mice has been linked to increases in EC proliferation [17, 20, 34, 45], which we also observed in our *Smad4* mutants (Fig. 2). Moreover, loss of *Smad4* led to an increase in EC cell size both in vivo and in vitro, which was unreported in *Alk1 *and *Eng* models (Fig. 3). These findings are supported by a recent study showing that loss of *Smad4 *caused an increase in the size and rates of proliferation of ECs in the coronary artery and under in vitro flow conditions [29]. Furthermore, work in zebrafish has shown that in response to increases in flow, *Eng*-deficient blood vessels enlarge [25]. Interestingly, our in vitro data suggest that, in *Smad4 *mutants, cell size changes may also occur in the absence of flow. Taken together, it appears that blood vessel enlargement in HHT models is affected not only by increases in EC proliferation but also by an increase in EC size itself. How these alterations may lead to AVM formation is unclear, although it is notable that defects in the NOTCH pathway (both gain-of-function and loss-of-function) cause AVM formation [40, 41, 46, 47] via an initial increase in size of ECs [48]. Whether AVMs in HHT patients form in a similar manner remains an open question, as evidence in zebrafish suggests that HHT-associated AVMs are not directly caused by alterations in NOTCH signaling [49].

**Table 1:** Comparison of HHT mouse models

Mutation	*Alkl*	*E* *ng*	*S* *mad4*
**Associated with**	HHT2	HHT1	HHT/JP
**Percentage of mutants with AVMs**	60% [20]	70% [17]	82%
**Angiogenic delay**	No reduction [20]	Reduced [1, 34]	Reduced
**Vessel size**			
Artery size		Enlarged [34]	Enlarged
Vein size	Enlarged [20]	Enlarged [17]	Enlarged
**Proliferation in**			
Artery		Increased [17, 34]	Increased
Vein	Increased [20]	Increased [17], not changed [34]	Increased
Capillary		Increased [17], not changed [34]	Increased
**Cell size**			Increased [29]^a^
**Smooth muscle coverage**	Increased [20]	Increased [17]	Increased
**Pericytes**	Decreased (only in capillaries) [20]		Decreased
**Artery identity**			
* Dll4*		No change [34]; not expressed in AVM	No change
* Ephrinb2*	Downregulated^??^	No change [17], downregulated [34]	No change
* Hey1*		Downregulated [34]	Downregulated
* Jagged1*	Downregulated [20, 24]	No change [17]; increased [34]	No change
* Notch1*	Downregulated [20, 24]		Downregulated
**Venous identity**			
* A* *pj*	No change [17]; upregulated [24]	No change (Expressed in AVM) [34]	No change; expressed in AVM
* Eph* *b* *4*		No change (Expressed in AVM) [34]	Reduced expressed in AVM
**VEGFR2 levels**	No change [24]	Altered VEGFA-induced kinetics [34]	Downregulated
**Respiratory distress**	24–48 h post-Tx Inj [20]		168–192 h post-Tx Inj

This table combines data from references using the inducible, endothelial-specific Cre-driver line Cdh5-Cre^ERT2^: Mahmoud et al. [17], Tual-Chalot et al. [20], Ola et al. [24], Jin et al. [34] and Poduri et al. [29]

^a^Note that Poduri et al. [29] used a ubiquitous *Rosa*-Cre^ER^ driver line. No in vitro data were included in this table

Nonetheless, our work demonstrated that expression of NOTCH signaling components, which are associated with arterial identity, as well as genes connected to venous and tip cell identity, are disrupted in the absence of *Smad4* (Fig. 4). We also revealed that these changes can occur in arteries, veins and/or capillaries; however, it is important to note that the AVMs themselves expressed all genes examined regardless of whether the marker was up- or downregulated in other vessel types. When comparing these results to those obtained in *Alk1* and *Eng* mouse models, we noted variations in AV gene expression between all three mutant backgrounds [17, 20, 24, 34, 38]. These differences could be due to tissue-specific effects related to the source tissues examined and/or the vascular expression patterns of *Alk1*, *Eng* and *Smad4*. For instance, some studies examined gene expression in isolated lung ECs [24], while others utilized brain and/or retinal ECs [20, 34]. Additionally, it is possible that expression levels in various vessel types play a role, as *Alk1* is highly expressed in arterial ECs [50], while *Eng* is only moderately expressed in arteries [51]. *Eng* also is expressed highly in capillaries and weakly in veins [52]. In comparison, *Smad4* is present in virtually all tissues [13, 14]. However, despite these differences, it is clear that overall disruptions in AV gene expression are consistent between all three mouse models of HHT. Further examination is needed to address whether alterations in AV identity are a primary cause or secondary effect of AVM formation. To this point, our work does not address whether the observed phenotypes and molecular changes are a cause or an effect of AVM formation, as experiments were performed after AVMs developed. This cause/effect relationship has not been explored in* Alk1* and *Eng *models of HHT either. Therefore, future studies addressing this issue will be important for identifying the underlying molecular defects that drive AVM pathogenesis versus those that are secondary effects of AVM formation.

It is also important to note that tamoxifen-inducible murine models of HHT have several limitations. HHT phenotypes arise in patients due to mutations (most commonly missense mutations) that lead to haploinsufficiency [10]. In contrast, mouse models of HHT often utilize null genetic backgrounds because loss of one allele of *Alk1*, *Eng* or *Smad4* does not result in consistent presence of AVMs in predictable locations [21, 26, 53–55]. Furthermore, HHT patients harbor germline mutations, which manifest from gestation and remain throughout adulthood. However, in mice, complete loss of *Alk1*, *Eng* or *Smad4* during gestation results in embryonic lethality making it impossible to study their postnatal impact on HHT [16, 27, 33, 56, 57]. For this reason, the mouse retina has become an effective model to study AVM formation; the retinal vasculature forms directly after birth allowing researchers to assess developmental angiogenesis, similar to vessel growth that would be seen in a developing human. Although these models do not perfectly mimic the genetic background of HHT patients, retinal AVMs form at consistent rates and locations providing a reliable model to investigate the mechanisms of AVM formation.

In our *Smad4*-iECKO retinas we noted delayed angiogenic outgrowth similar to *Eng* mutants [17, 34], while *Alk1* mutant retinas did not exhibit reduction in vascular outgrowth [20]. Interestingly, our *Smad4*-iECKO mice exhibit a significant reduction in *Eng* transcript levels but show no changes in *Alk1* mRNA levels (Fig. 5A). This could account for the observed similarities in reduced vascular outgrowth between *Smad4* and *Eng*, but not *Alk1* mice. However, this result also illustrates the complex association between the TGFβ pathway and HHT, as *Eng* expression levels are reduced in the *Alk1* mouse models of HHT [20, 24], yet show no changes in outgrowth. To our knowledge, it is unknown what happens to levels of *Alk1* expression in the *Eng *HHT model, or whether *Smad4 *levels are affected in either *Alk1* or *Eng* mouse models. Moving forward, it will be important to understand the association between *Alk1*, *Eng* and *Smad4* in HHT because even though it is expected that all three cooperate in a linear manner in the TGFβ pathway, differences in phenotypes (Table 1) suggest this might not be the case.

The overall objective of our work was to develop a *Smad4* model of HHT that could be used to identify the TGFβ targets that drive AVM formation, as almost nothing is known about these downstream effectors. To this end, we explored a possible link with the vascular endothelial growth factor (VEGF) signaling pathway that has been previously suggested in other HHT models [21, 24, 26, 34, 42, 53]. For instance, homozygous-induced deletion of *Alk1* or* Eng* in adult mice requires the presence of exogenous VEGF before AVMs will form in the brain, suggesting that activation of the VEGF pathway is needed for AVM formation [21, 26]. To this end, VEGF neutralizing antibodies have been shown to prevent wound-induced skin AVMs from developing in *Alk1*-deficient mice [53]. Furthermore, in the absence of *Alk1* and *Eng*, several studies have reported increased *Vegfr2* expression and altered VEGFR2 kinetics in vitro [24, 34, 42]. In contrast, our data showed that loss of *Smad4* led to a reliable and significant decrease in *Vegfr2* expression both in vitro and in vivo (Fig. 6). This is consistent with a previous study on human patients with cerebral brain AVMs where there was a marked decrease in *Vegfr2* expression [58]. Contrary to other HHT studies, the reduction of *Vegfr2* in *Smad4*-iECKO mice could potentially be attributed to the downregulation in *Nrp1*, a VEGFR2 co-receptor. Studies have shown that decreased *Nrp1* levels correlate with reduced *Vegfr2* expression [59, 60]. Although other HHT studies did not find reduced *Vegfr2* levels, homozygous deletion of both *Smad4* and *Vegfr2* produced similar results to those obtained in double *Alk1*- and *Vegfr2*-deficient retinas [24]. In each study, deletion of both alleles of *Vegfr2* in the *Alk1* or *Smad4* null backgrounds resulted in inhibition of retinal vascular development, suggesting that appreciable loss of *Vegfr2* in the absence of either *Alk1* or *Smad4* overrides HHT-like phenotypes because the vasculature is severely underdeveloped (Fig. 7). We did note that AVMs still formed in both experiments at fewer and similar rates in *Alk1* and *Smad4* mutants, respectively. However, in further studies we demonstrated that loss of a single *Vegfr2* allele in the *Smad4* mutant background led to an enhancement of vascular phenotypes associated with *Smad4*-iECKO retinas; the vascular front exhibited a consistent increase in density and AVMs showed a substantial enlargement. Alternatively, increased AVM size could be attributed to altered blood flow rates, hemodynamics forces and/or rates of oxygen diffusion caused by the overall stunted growth of the mutant blood vessels, rather than due to the loss of VEGFR2 directly. Future studies will be needed to understand how these processes are altered in TGFβ mutant backgrounds and how those contributions may affect severity of AVMs.

This SMAD4-VEGFR2 association is somewhat contrary to the clinical use of bevacizumab (also known as Avastin), which is a humanized anti-VEGF monoclonal antibody that sequesters VEGF to prevent it from binding both VEGFR1 and VEGFR2 subsequently hindering angiogenesis [61, 62]. Bevacizumab is currently used as a palliative therapy for HHT where it alleviates symptoms such as chronic nosebleeds but is not considered a long-term therapy [63]. Studies on the use of bevacizumab have been performed in mature vascular networks, namely that of adult humans and mice [64, 65]. Little information is known about the effects of bevacizumab in children or developing/remodeling vascular networks. Our work suggests that the connection between SMAD4 and VEGFR2 is different during developmental angiogenesis, when AVMs are thought to form, as compared to mature, established vascular networks. Therefore, further research on the effects of bevacizumab in developing vascular networks is needed, as our results indicate that bevacizumab may enhance developmental HHT phenotypes.

## Materials and methods

### Mice

All animal experiments were performed in accordance with Tulane University’s Institutional Animal Care and Use Committee policy. To create our *Smad4*-iECKO mouse model, we crossed an endothelial-specific, tamoxifen-inducible Cre-driver line (Tg(Cdh5-Cre^ERT2^)^1Rha^, further referred to as *Cdh5*-Cre^ERT2^) [30] with a conditional Smad4 mouse (Smad4^f/f^) [31]. To confirm that *Smad4* was being knocked out only in ECs, we mated* Smad4*-iECKO mice with a *Rosa26*-EYFP reporter mouse (Gt(ROSA)26Sor^tm1(EYFP)Cos^) [32]. Induction of tamoxifen was done using 0.075 mg tamoxifen (Sigma T5648) per gram of body weight on postnatal days 1 and 4. Note: For *Vegfr2*-iECKO mice only one injection of Tx was given on P1. For experiments, *Smad4*^f/f^;*Cdh5*-Cre^ERT2^ (otherwise referred to as *Smad4*-iECKO) mice were the experimental group, while *Smad4*^f/f^ littermates were used as controls. Genotyping primers and conditions can be found in supplemental methods.

### Hematoxylin and eosin staining of murine lungs

Neonatal lungs were dissected from postnatal day 8 pups and fixed for 4 h in 4% PFA at 4 °C. The lungs were then embedded in paraffin and sectioned at 10 µm. Sections were washed in xylenes twice then put through a rehydration series. Slides were placed into hematoxylin solution for ~ 1 min and then rinsed with water for several minutes. This process was repeated for eosin staining. Slides were then mounted with Permount (Thermo).

### Retinal whole mount stains

Retinas were dissected and stained as previously described [66]. The following antibodies were used at a 1:100 concentration: αSMA (Sigma C6198), cleaved CASPASE-3 (Cell signaling 9661), COLLAGEN IV (Millipore AB756P), ENDOMUCIN (Santa Cruz 6415), ERG (Abcam 92513), GFP (Aves GFP-1020), KI67 (Cell Signaling 9449), NG2 (Millipore 5320), PECAM/CD31 (BD 553370), VEGFR2 (BD 555307). Additionally, the following immunofluorescent stains were performed according to the manufacturer’s instructions: Dapi (Life Technologies R37606), Isolectin-488 (Invitrogen 21411), Isolectin-594 (Invitrogen 21413), Isolectin-647 (Invitrogen 32450). Confocal images were taken at the same exposure settings for both mutant and control retinas, so fluorescent intensity could be compared.

### In situ hybridizations

In situ hybridizations were performed as previously described [66]. In situ hybridizations were performed in batches where mutants and controls were subjected to the colorimetric reaction for the same period of time so that results could be compared. The following probes were synthesized from plasmids containing: *Apelin* (Dharmacon), *Apj* (Dharmacon), *Ephb4*, *Notch4*. Images were taken using a Leica M205 FA stereomicroscope.

### Isolation of endothelial cells

Retinal and lung endothelial cell isolation were performed as previously described [67]. Briefly, tissue (either lung or retina) was digested in a collagenase/dispase solution and minced into fine pieces. After obtaining a single cell suspension, sheep anti-rat IgG dynabeads (Invitrogen 11035) coated with PECAM/CD31 antibody (BD 553370) were used to isolate endothelial cells. Cells were either used for RNA collection immediately or allowed to grow in EGM-2 medium for one week before being used for protein or RNA collection.

### qPCR and analysis

All quantitative real-time PCR (qPCR) experiments were performed using RNA isolated with a GeneJET RNA Purification Kit (Thermo K0732) and quantified using a Nanodrop (Thermo). For each sample, 500–1000 ng of RNA was used for cDNA synthesis using a iScript cDNA Synthesis Kit (Bio-Rad). qPCRs were run using SYBR green mastermix (Thermo K0221) on a Bio-Rad CFX96 Touch Real-Time PCR Detection machine. Analysis was performed using the double delta Ct method, and statistics were generated using GraphPad Prism. For all qPCR experiments, three independent biological replicates were used and three technical replicates were performed per sample. Primers were verified for specificity and efficiency and can be found in supplemental materials.

### Quantification of retinal images

All retina images were analyzed using Nikon NIS-Elements AR Analysis 64-bit software, and ImageJ software was used to measure vascular outgrowth, cell area and cell density.

### Statistical analysis

GraphPad Prism software was used for all statistical analysis. For all statistics, sample size (*n*) indicates the number of independent biological samples. A minimum of three technical replicates was included per sample. For statistical analysis, we ran unpaired two-tailed Student’s *t* test where a *p* value of < 0.05 was considered significant.

## Electronic supplementary material

Below is the link to the electronic supplementary material.

Supplemental Figure 1 Lung defects are associated with postnatal endothelial loss of* Smad4*. (**A**, **B**). Hematoxylin and eosin stained lung sections from *Smad4*^f/f^ control (*n* = 3) and *Smad4*-iECKO (*n* = 3) postnatal day 8 pups. (**A’**, **B’**) Magnified views of dotted boxes in **A** and **B** show blood filled regions in the lungs of *Smad4* mutants that are absent in *Smad4* control lungs. Scale bars: 250 µm. (**C**–**D’**) Immunofluorescent images of *Smad4*^f/f^;*Rosa26*-EYFP (*n* = 8) and *Smad4*-iECKO;*Rosa26*-EYFP (*n* = 7) P7 retinas injected with Tx at P1 and P4, and stained for EYFP (green; anti-GFP antibody) and Isolectin-IB4 (red). The *Rosa26*-EYFP reporter line only expresses enhanced yellow fluorescent protein (EYFP) in the presence of Cre-recombinase. Notice efficient, EC-specific expression of EYFP in *Smad4* mutants but not in controls lacking *Cdh5*-Cre^ERT2^. Scale bar represents 200 µm. (PDF 2457 kb)

Supplemental Figure 2 Various morphologies are associated with *Smad4* AVMs and are less severe or absent when tamoxifen is administered after postnatal day 1. (**A**–**H**) Close up views of P7 *Smad4* mutant retinas stained with Isolectin-IB4 (IB4; green), which marks all blood vessels. Arrows mark AVMs. (**I**–**K**) Confocal images of *Smad4*-iECKO, IB4 stained P7 retinas from pups given a single Tx injection at P1 (I), P2 (J) and P3 (**K**). Notice the loss of *Smad4* associated vascular defects, such as AVMs and vessel outgrowth (dotted circles), as Tx is injected at later neonate stages. Arteries, A; veins, V. (PDF 11436 kb)

Supplemental Figure 3 *Smad4* depletion leads to migration defects in scratch wound healing assays. (**A**–**B’**) Scratch assay results of nonsilencing-shRNA control (**A**, **A’**) and Smad4-shRNA (**B**, **B’**) C166 cells at 0 (**A**, **B**) and 23 (**A’**, **B’**) hours post scratch. Dotted black lines outline EC-free areas. Note the significantly reduced repopulation of the wound in Smad4-shRNA C166 cells compared to control nonsilencing-shRNA. (**C**) Quantification of EC-free areas as assessed with Image J. (**D**) qPCR analysis of *Smad4* gene expression in nonsilencing-shRNA versus Smad4-shRNA C166 cells reveals ~60% reduction in *Smad4* transcript levels. (PDF 4128 kb)

Supplemental Figure 4 Variability of vessel density at the vascular front of *Smad4*-iECKO retinas. (**A**–**F**). Whole retina confocal images of *Smad4*-iECKO mice. White arrows denote AVMs. Scale bar represents 500 µm (PDF 3669 kb)

Supplementary material 5 (DOCX 33 kb)
