# Supplementary figures and images for: Vascular deficiency of Smad4 causes arteriovenous malformations: a mouse model of Hereditary Hemorrhagic Telangiectasia
Source: Angiogenesis. 2018 Feb 19;21(2):363–80. doi: 10.1007/s10456-018-9602-0 (PMC5878194; doi:10.1007/s10456-018-9602-0)

*Smad4<sup>fl/f</sup>*

*Smad4-iECKO*

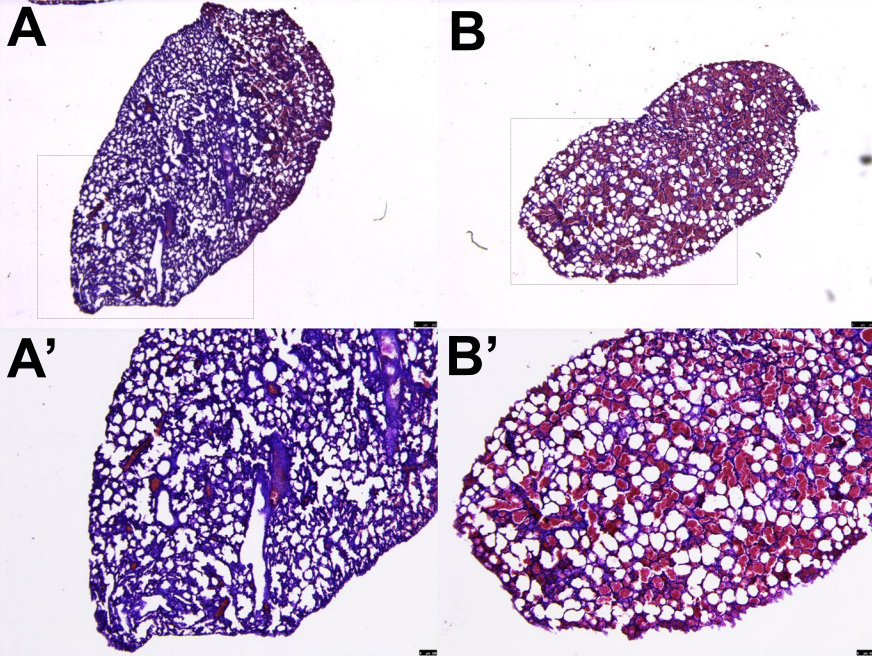

*Smad4<sup>fl/f</sup>;Rosa26-EYFP* *Smad4<sup>fl/f</sup>-iECKO;Rosa26-EYFP*

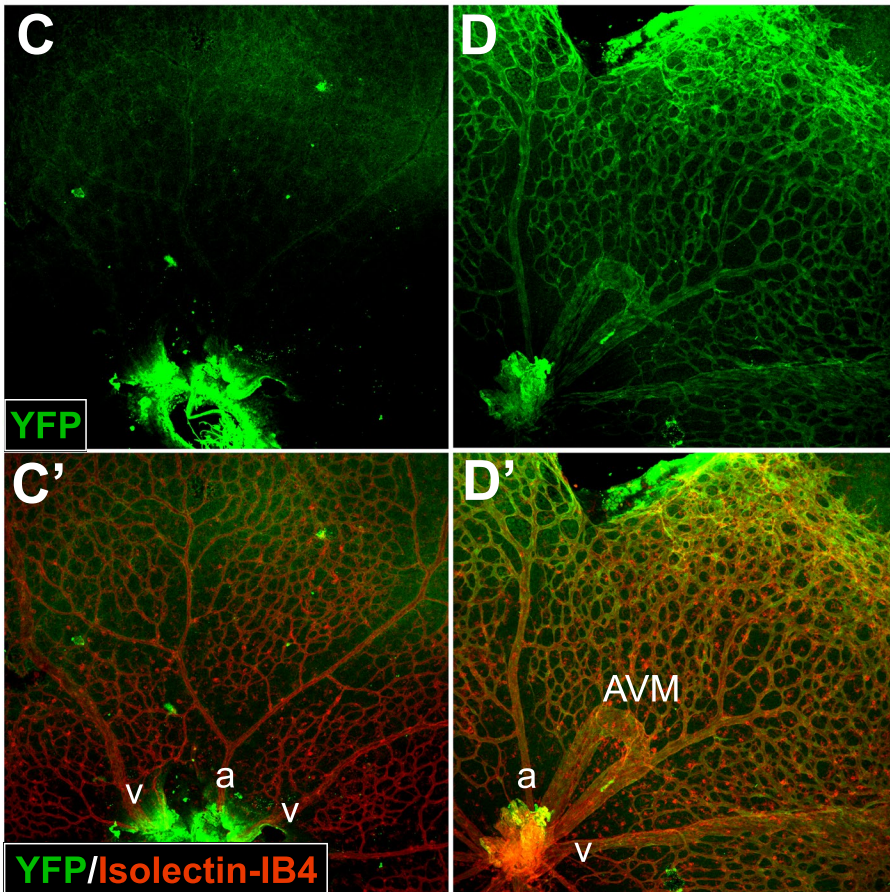

Supplement: Supplementary file 1 — Lung defects are associated with postnatal endothelial loss of Smad4. (A, B). Hematoxylin and eosin stained lung sections from Smad4f/f control (n = 3) and Smad4-iECKO (n = 3) postnatal day 8 pups. (A’, B’) Magnified views of dotted boxes in A and B show blood filled regions in the lungs of Smad4 mutants that are absent in Smad4 control lungs. Scale bars: 250 µm. (C–D’) Immunofluorescent images of Smad4f/f;Rosa26-EYFP (n = 8) and Smad4-iECKO;Rosa26-EYFP (n = 7) P7 retinas injected with Tx at P1 and P4, and stained for EYFP (green; anti-GFP antibody) and Isolectin-IB4 (red). The Rosa26-EYFP reporter line only expresses enhanced yellow fluorescent protein (EYFP) in the presence of Cre-recombinase. Notice efficient, EC-specific expression of EYFP in Smad4 mutants but not in controls lacking Cdh5-CreERT2. Scale bar represents 200 µm. (PDF 2457 kb) [file 10456_2018_9602_MOESM1_ESM.pdf]

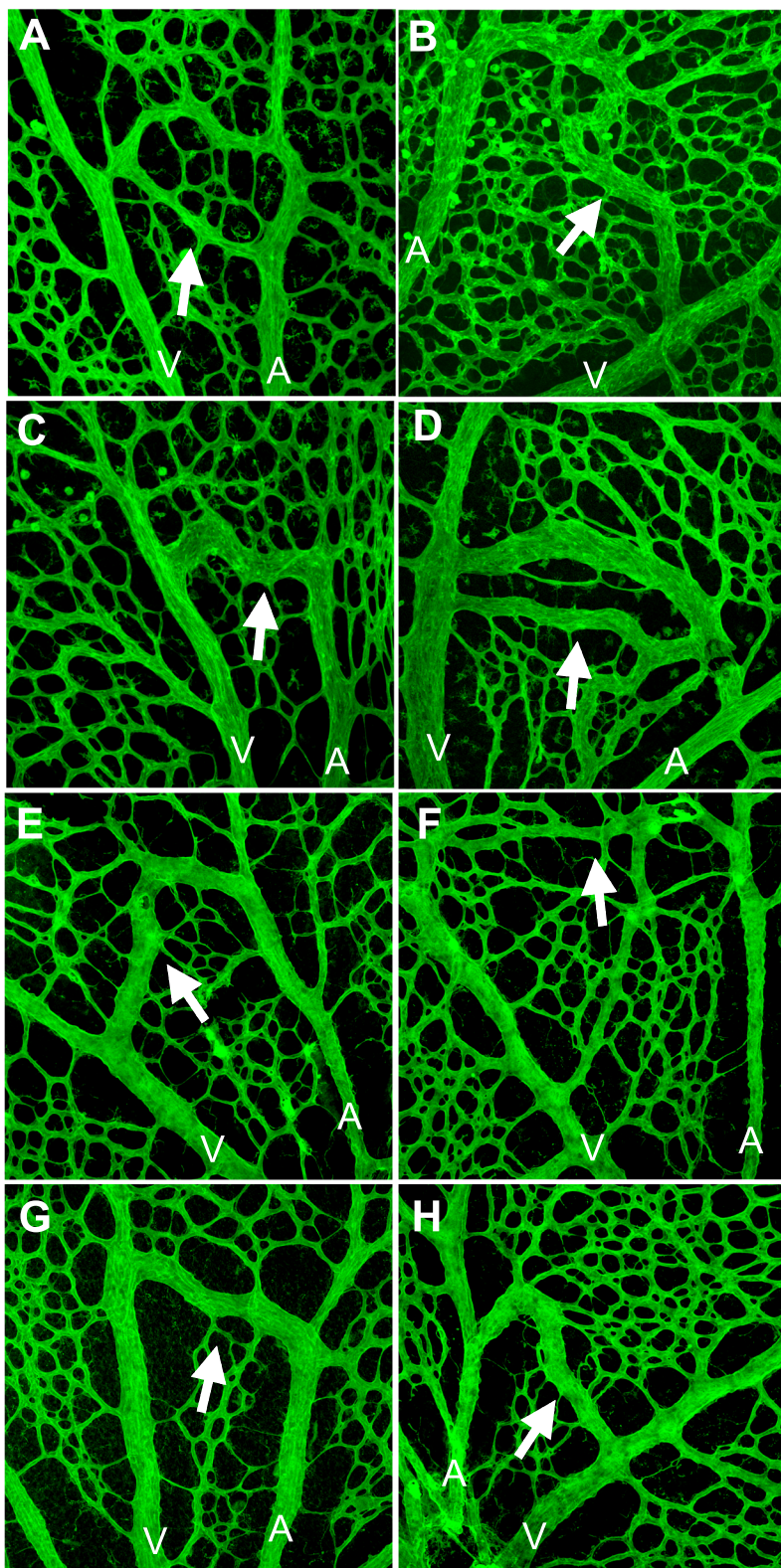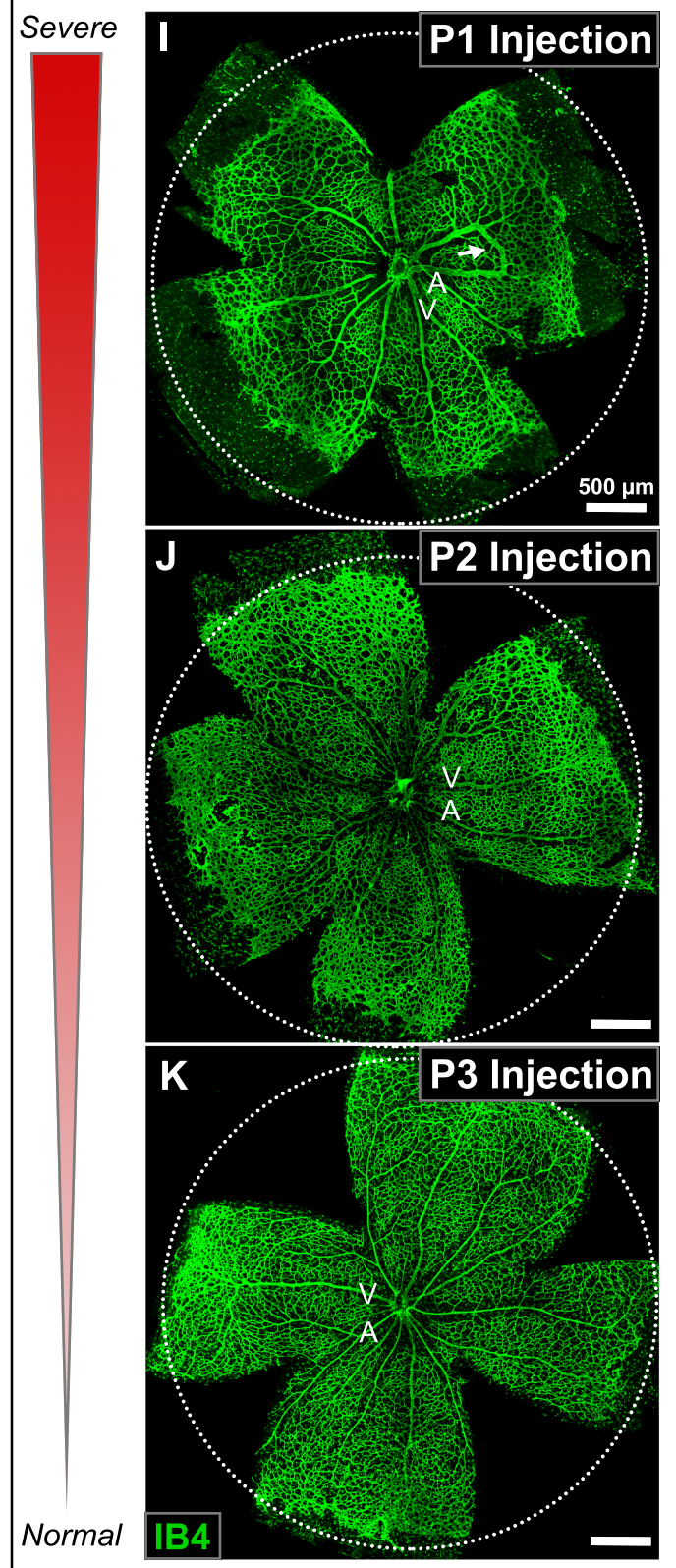

Supplement: Supplementary file 2 — Various morphologies are associated with Smad4 AVMs and are less severe or absent when tamoxifen is administered after postnatal day 1. (A–H) Close up views of P7 Smad4 mutant retinas stained with Isolectin-IB4 (IB4; green), which marks all blood vessels. Arrows mark AVMs. (I–K) Confocal images of Smad4-iECKO, IB4 stained P7 retinas from pups given a single Tx injection at P1 (I), P2 (J) and P3 (K). Notice the loss of Smad4 associated vascular defects, such as AVMs and vessel outgrowth (dotted circles), as Tx is injected at later neonate stages. Arteries, A; veins, V. (PDF 11436 kb) [file 10456_2018_9602_MOESM2_ESM.pdf]

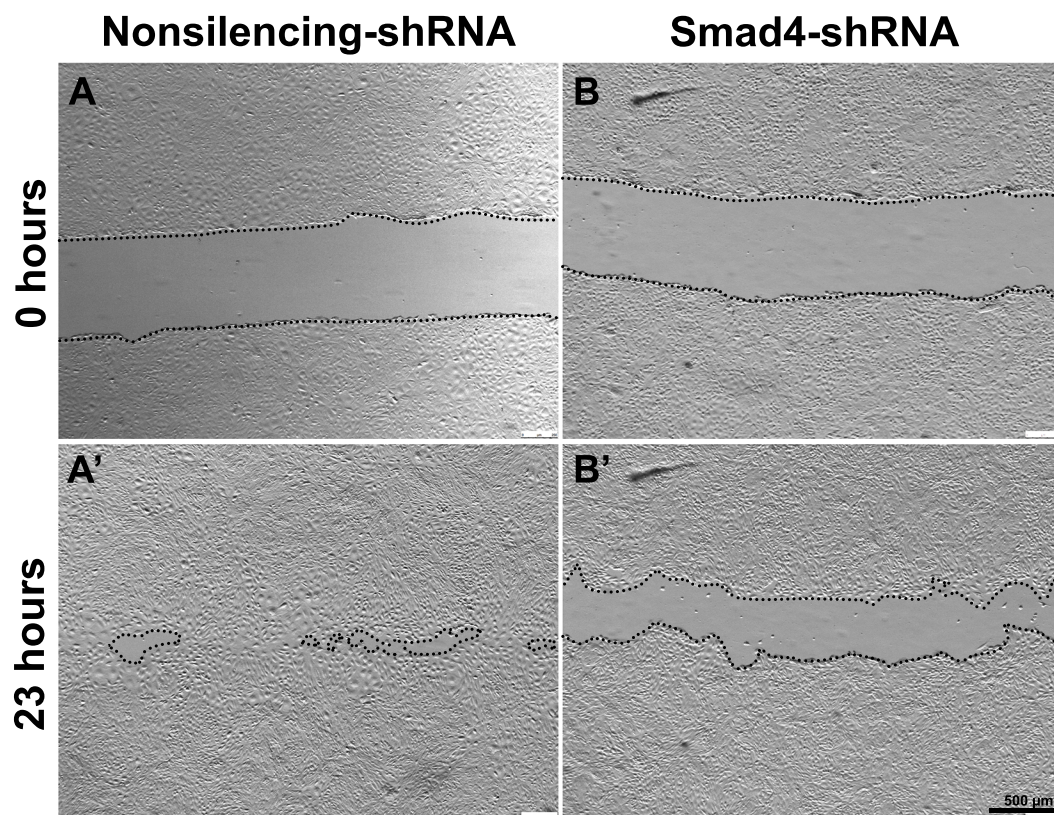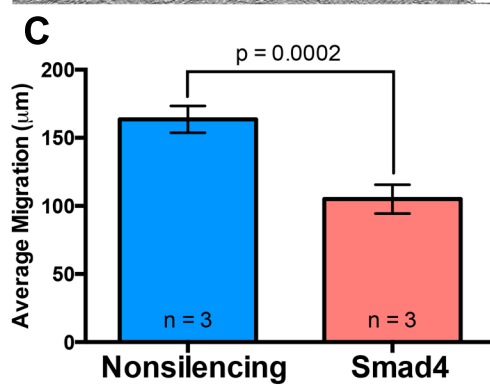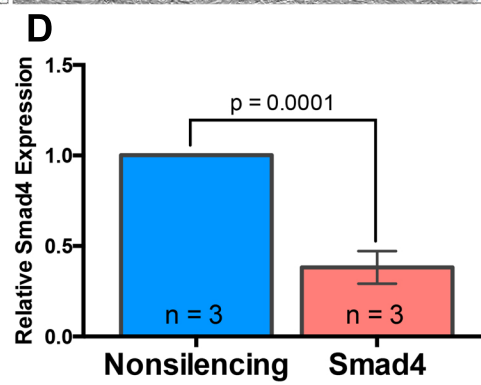

Supplement: Supplementary file 3 — Smad4 depletion leads to migration defects in scratch wound healing assays. (A–B’) Scratch assay results of nonsilencing-shRNA control (A, A’) and Smad4-shRNA (B, B’) C166 cells at 0 (A, B) and 23 (A’, B’) hours post scratch. Dotted black lines outline EC-free areas. Note the significantly reduced repopulation of the wound in Smad4-shRNA C166 cells compared to control nonsilencing-shRNA. (C) Quantification of EC-free areas as assessed with Image J. (D) qPCR analysis of Smad4 gene expression in nonsilencing-shRNA versus Smad4-shRNA C166 cells reveals ~60% reduction in Smad4 transcript levels. (PDF 4128 kb) [file 10456_2018_9602_MOESM3_ESM.pdf]

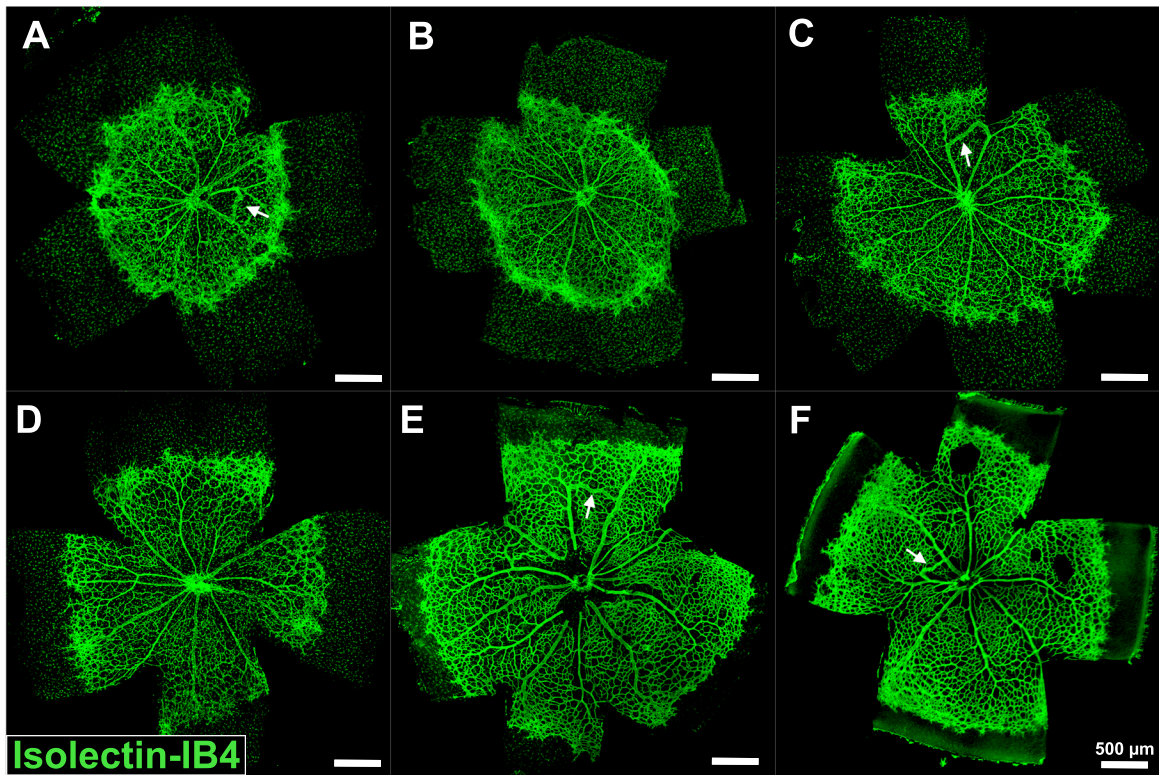

Supplement: Supplementary file 4 — Variability of vessel density at the vascular front of Smad4-iECKO retinas. (A–F). Whole retina confocal images of Smad4-iECKO mice. White arrows denote AVMs. Scale bar represents 500 µm (PDF 3669 kb) [file 10456_2018_9602_MOESM4_ESM.pdf]
