# Supplementary material for: Vascular deficiency of Smad4 causes arteriovenous malformations: a mouse model of Hereditary Hemorrhagic Telangiectasia
Source: Angiogenesis. 2018 Feb 19;21(2):363–80. doi: 10.1007/s10456-018-9602-0 (PMC5878194; doi:10.1007/s10456-018-9602-0)
Supplement: Supplementary file 5 — Supplementary material 5 (DOCX 33 kb) [file 10456_2018_9602_MOESM5_ESM.docx]

**Supplemental Methods**

**Genotyping**

Genotyping was run using the following PCR Conditions: 94°C for 2 min then 36 cycles of 94°C for 35 sec, 60°C for 35 sec, 72°C for 1 min followed by 72°C for 5 min using the following primers:

| **Gene** | **Forward Primer** | **Reverse Primer** | **WT (bp)** | **Flox (bp)** | |
| --- | --- | --- | --- | --- | --- |
| Cre | GATCGCTGCCAGGATATACG | CATCGCCATCTTCCAGCAG |  | | 572 |
| Smad4 | TAAGAGCCACAGGGTCAAGC | TTCCAGGAAAAACAGGGCTA | 436 | | 500 |
| YFP | CCTGAAGTTCATCTGCACCA | TGCTCAGGTAGTGGTTGTCG |  | | 478 |
| VEGFR2 | TGGAGAGCAAGGCGCTGCTAGC | CTTTCCACTCCTGCCTACCTAG | 322 | | 439 |

**Quantitative Polymerase Chain Reaction (qPCR)**

Primers used in qPCR experiments were as follows:

| **Gene** | **Forward Primer** | **Reverse Pirmer** |
| --- | --- | --- |
| Acta2 | GTCCCAGACATCAGGGAGTAA | TCGGATACTTCAGCGTCAGGA |
| Alk1 | GGGCCTTTTGATGCTGTCG | TGGCAGAATGGTCTCTTGCAG |
| Apelin | GGCCTTCTCCGTCTTTGTCG | CCCTCTTGTGCTTCTATCTCTCC |
| APJ | GGTTACAACTACTATGGGGCTGA | AGCTGAGCGTCTCTTTTCGC |
| CoupTFII | TCAACTGCCACTCGTACCTG | CCATGATGTTGTTAGGCTGCAT |
| Cx40 | GGTCCACAAGCACTCCACAG | CTGAATGGTATCGCACCGGAA |
| Dll4 | CAGGGACAAGAATAGCGGCA | GTTTCCTGGCGAAGTCTCTGG |
| Desmin | TCATCGCCCTTCCCCTTC | GTCCACAAACTCGGTCCTG |
| Endomucin | AATACCAGGCATCGTGTCAGT | CTGATTCTCAGTCTTGTTCTGGG |
| Eng | TTCTCACACACGTGGCCC | CCGATGCTGTGGTTGGTACT |
| EphrinB2 | GAGTGCGCAGAACTGGGA | CCGGGTAGAAATTTGGAGTTCG |
| Hey1 | TTAACTCCTCCTTGCCCGCC | ACTCAAGTTTCCATTCTCGTCCG |
| Hey2 | AAGCGCCCTTGTGAGGAAAC | GGTAGTTGTCGGTGAATTGGAC |
| Jagged1 | CCTCGGGTCAGTTTGAGCTG | CCTTGAGGCACACTTTGAAGTA |
| Ng2 | GGGCTGTGCTGTCTGTTGA | TGATTCCCTTCAGGTAAGGCA |
| Notch1 | GGATGACCTAGGCAAGTCGG | CCAGCAACACTTTGGCAGTC |
| Notch4 | CTCTTGCCACTCAATTTCCCT | TTGCAGAGTTGGGTATCCCTG |
| Nrp1 | GACAAATGTGGCGGGACCATA | TGGATTAGCCATTCACACTTCTC |
| Nrp2 | GCTGGCTACATCACTTCCCC | CAATCCACTCACAGTTCTGGTG |
| PECAM/CD31 | CTGCCAGTCCGAAAATGGAAC | CTTCATCCACCGGGGCTATC |
| Vegfr2 | CTAGCTGTCGCTCTGTGGTTC | GCTGTCCCCTGCAAGTAATCTGA |
| ZO1 | GCCGCTAAGAGCACAGCAA | TCCCCACTCTGAAAATGAGGA |
| ODC | TGACTGTGCAAGCAAGACTGA | TGTGCTCTGGCGACTTTCAT |
